# Association of quality and quantity of macronutrients intake with obesity, new anthropometric indices, lipid accumulation, and blood lipid risk index in Tehranian women

**DOI:** 10.1002/fsn3.3991

**Published:** 2024-02-04

**Authors:** Zahra Salehi, Pegah Rahbarinejad, Batoul Ghosn, Leila Azadbakht

**Affiliations:** ^1^ Department of Community Nutrition School of Nutritional Sciences and Dietetics, Tehran University of Medical Sciences Tehran Iran; ^2^ Diabetes Research Center Endocrinology and Metabolism Clinical Sciences Institute, Tehran University of Medical Sciences Tehran Iran; ^3^ Department of Community Nutrition School of Nutrition and Food Science, Isfahan University of Medical Sciences Isfahan Iran

**Keywords:** carbohydrate, cardiovascular risk factors, fat, protein

## Abstract

**Background:**

This study examines the association between micronutrient intake, anthropometric indices, lipid accumulation, and blood lipid risk index among Tehranian women.

**Methods:**

A cross‐sectional study was conducted on 556 Tehranian women. Dietary intake was measured using a Food Frequency Questionnaire. Biochemical assessment and anthropometric indices were measured, and demographic information and physical activity were collected.

**Results:**

Participants with the highest intake of carbohydrates were more prone to obesity. Conversely, those in the top tertile for protein intake had a lower likelihood of obesity and higher levels of lipid accumulation product (LAP). The highest fat consumers had a 63% decreased chance of having a high Castelli's Risk Index 1 (CRI‐1). A higher glycemic index (GI) and glycemic load (GL) were linked to an increased probability of a high atherogenic coefficient (AC). Women in the top tertile of GL were significantly more likely to be obese and had lower odds for high LAP. Participants in the top tertile of aromatic amino acids/branched chain amino acids (AAA/BCAA) had significantly lower chances of high CRI‐1 and a high atherogenic index of plasma (AIP). Those in the highest tertile of monounsaturated fatty acids/polyunsaturated fatty acids (MUFA/PUFA) had lower odds of obesity and high AIP.

**Conclusions:**

The amount of carbohydrate (g) and protein intake (%), dietary GL, and the ratio of MUFA to PUFA were associated with obesity. The amount of fat intake (g) and AAA/BCAA indices were associated with CRI‐1. LAP decreased with an increase in GL. AC increased with an increase in GI and GL. AAA/BCAA and MUFA/PUFA were associated with AIP.

## Graphical abstract

In the present study, we investigated the relationship between the quality and quantity of macronutrients in the diet, with obesity, new anthropometric indices, lipid accumulation, and blood lipid risk index. We found that carbohydrate (g) and protein intake (%), dietary glycemic load (GL), and the ratio of monounsaturated fatty acids to polyunsaturated fatty acids (MUFA/PUFA) are associated with obesity. The amount of fat intake (g) and the indices of aromatic amino acids to branched chain amino acids (AAA/BCAA) are associated with Castelli's Risk Index‐1 (CRI‐1). Lipid accumulation product (LAP) decreases with an increase in GL, while the atherogenic coefficient (AC) increases with an increase in both glycemic index (GI) and GL. AAA/BCAA and MUFA/PUFA are associated with the atherogenic index of plasma (AIP).

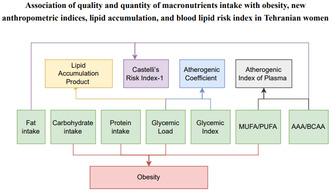

## 1 INTRODUCTION

Many studies have shown an increase in the prevalence of obesity, abdominal obesity, and lipid accumulation among people around the world which is one of the causes of cardiovascular diseases and type 2 diabetes (Esmaillzadeh et al., 2005; Reaven, 1988). Should the current trajectory of escalating overweight and obesity rates persist, projections suggest that by 2030, nearly 57.8% of adults globally could be classified as either overweight or obese, as indicated by prior research (Kelly et al., 2008; Santiago et al., 2015). The obesity prevalence in Iran is higher in women than men (Alberti et al., 2009; Azizi et al., 2003; Basagoudar & Chandrashekhar, 2013; Cameron et al., 2004; Ghassemi et al., 2002). The increasing body of evidence suggests that the accumulation of fat in the abdomen significantly contributes to cardiovascular risk factors. This has led to the need for new anthropometric measures to evaluate the storage of abdominal fat. In response to this need, Krakauer and colleagues, as well as Tomas and his team, introduced the body shape index (ABSI) and the body roundness index (BRI), respectively, as estimators of body fat distribution (Perona et al., 2019). The body roundness index (BRI) is a novel anthropometric measure designed to estimate both the percentage of body fat and visceral adipose tissue (Rico‐Martín et al., 2020). Abdominal volume index (AVI) is a reliable and easy anthropometric tool for estimating obesity that is strongly associated with impaired glucose tolerance and diabetes (Guerrero‐Romero & Rodríguez‐Morán, 2003). Lipid accumulation product index (LAP), calculated from waist circumference (WC) and serum triglyceride (TG), is suggested as a better predictor of visceral fat (Ray et al., 2018). Recent research suggests that indices derived from individual plasma lipid levels, such as the Atherogenic Index of Plasma (AIP), Castelli's Risk Index‐1 (CRI‐1), and the Atherogenic Coefficient (AC), offer more dependable forecasts of events related to cardiovascular disease (CVD) compared to conventional fat measurements (Culha et al., 2020). In recent years, a new factor called hypertriglyceridemia waist phenotype has also been introduced. This index includes insulin resistance with increased serum levels of apolipoprotein B and an increase in the number of small low‐density lipoproteins (LDLs) (Lamarche et al., 1998; Lemieux et al., 2000).

Although genetic factors contribute to obesity, dietary intakes, and lifestyle are modifiable risk factors that serve a crucial function in both the prevention and management of these conditions. Dietary intake plays an important role in obesity, waist circumference value, and serum triglyceride levels as well as hypertriglyceridemia index (Wycherley et al., 2012). Different amounts and sources of fat (Lamarche et al., 1998; Lemieux et al., 2000), carbohydrates (Aller et al., 2011; Kahn & Valdez, 2003), and proteins (Kahn & Valdez, 2003) are effective in the treatment and prevention of obesity, especially abdominal obesity. However, there is still disagreement in this regard. Although many studies have examined the role of macronutrients and food composition in weight changes, recent research has prioritized the impact of macronutrient quality and its relationship with disease over the quantity of macronutrient intake.

In the field of macronutrient quality and obesity, the only available study is the study of Kim et al. (2018) which showed that a higher carbohydrate quality index (CQI) is negatively correlated with the obesity prevalence. Actually, most studies have focused on the number of macronutrients in obesity. The connection between dietary habits and the factors mentioned is not well understood. Therefore, this study aims to explore the link between the quality and quantity of carbohydrate, protein, and fat consumption and the prevalence of obesity and abdominal obesity, hypertriglyceridemia waist phenotype, new anthropometric indicators, and new risk factors related to fat status in women referring to health centers in Tehran.

## 2 MATERIALS AND METHODS

### 2.1 Participant selection

This cross‐sectional study was conducted on a sample of 556 women, aged between 20 and 50. The participants were chosen using a cluster random sampling technique. The study included women who visited 10 health centers in southern Tehran, Iran, during the period from 2017 to 2018. The estimated number of women sampled from each health center depended on the proportion of the total number of women visiting each health center. Women qualified to participate in the study were chosen according to these criteria: Age 20–50 years, agreeing to participate in the project, being Iranian, not immigrant, not pregnant and breastfeeding, not suffering from diseases such as diabetes, cardiovascular disease, cancer, liver and kidney diseases, and not taking drugs that affect fat and glucose metabolism or blood pressure. Lack of cooperation was a measure of exclusion from the study. Furthermore, women with extreme energy intake (either less than 500 or more than 3500 kcal per day) were excluded from the analysis. The sample size was calculated with weight as the primary dependent variable, considering an *α* value of 0.05 and a *β* value equivalent to 2% of the median weight (Fleiss, 1981). Then, by setting P1 = 18%, P2 = 8%, and R = 30/70, the number of participants calculated was 445 (Fuente‐Arrillaga et al., 2014). Finally, this sample number was multiplied by 1.25 due to its clustering, and 556 women were chosen to be included. Because no over‐ or under‐reporting of total energy was observed, no participant have been excluded. Conscious written consent was obtained from all study participants. The study protocol was confirmed by the National Institute for Medical Research Development (Nimad) with code 995396.

### 2.2 Dietary assessment and quality and quantity of macronutrients calculation

A 168‐item food frequency questionnaire (FFQ) was used through face‐to‐face interviews to assess participants' nutritional habits. The reliability and validity of this questionnaire have been proven in previous research (Asghari et al., 2012; Esfahani et al., 2010; Mirmiran et al., 2010). The Food Frequency Questionnaire (FFQ) incorporated portion sizes categorized according to the frequency (daily, weekly, or monthly) of food consumption over the past year. The portion size of all foods consumed by each individual was converted from household measurements to grams. To compute the energy and nutrients average intake collected via the FFQ, Nutritionist IV updated version software was used, which was modified for Iranian food (version 7; N‐Squared Computing, Salem, OR, USA).

The amount of carbohydrates, proteins, and fats were evaluated as a percentage of calories per day and grams per day (Byun et al., 2019; Ciuris et al., 2019; Cordner & Tamashiro, 2015; Li et al., 2016; Smith et al., 1994). We used glycemic index (GI) and glycemic load (GL) (Murakami et al., 2008) to assess carbohydrate quality. Protein quality was measured using tertiles of received cyclic amino acid to tertiles of branched chain amino acid (Dejong et al., 2007), and fat quality obtained by calculating the ratio of monounsaturated fatty acid (MUFA) to polyunsaturated fatty acid (PUFA) (Daza et al., 2005), and saturated fatty acid (SFA) to PUFA (Chechi & Cheema, 2006).

### 2.3 Biochemical assessment

Biochemical parameters were measured after 12 h of night fasting. On the day of blood collection, fasting blood sugar (FBS) was determined, and the remaining serum was stored at −80°C for further analysis. Serum levels of total cholesterol (TC), low‐density lipoprotein cholesterol (LDL‐C), high‐density lipoprotein cholesterol (HDL‐C), triglycerides (TG), and FBS were assessed using commercially available enzymatic reagents (Pars Azmoun, Tehran, Iran) which is equivalent to an automated analysis system (Selectra E, Vitalab, Holliston, The Netherlands). Also, lipid accumulation product (LAP) (Rotter et al., 2017), blood lipids risk index with Castelli's risk index‐1 (CRI‐1), atherogenic coefficient (AC), and atherogenic index of plasma (AIP) components (Culha et al., 2020), which are made based on lipid profile, were evaluated. The formulas are as follows:

$$\text{Female}\;\mathrm{LAP}=\left[\text{waist}\;\left(\mathrm{cm}\right)-58\right]\times \mathrm{TG}\;\text{concentration}\;\left(\text{mmol}/L\right)$$

$$\mathrm{CRI}-1=\frac{\mathrm{TC}}{\mathrm{HDL}}\mathrm{AC}=\text{non}-\frac{\mathrm{HDL}}{\mathrm{HDL}}$$

$$\mathrm{AIP}=\log10\;\left(\mathrm{TGs}/\mathrm{HDL}\right)$$

### 2.4 Anthropometric measurements

Usual anthropometric indicators (height, weight, and WC) and novel anthropometric judgments were measured. Measurement of participants' body weight was done using a digital scale (Seca725 GmbH & Co., Hamburg, Germany) to the nearest 0.1 kg, with participants wearing the least amount of clothing. The height was determined using an inflexible tape with an accuracy of nearly 0.5 cm. Prior to the measurements, participants were instructed to stand shoeless and maintain a normal shoulder position against the wall. The WC was taken at the narrowest level on light clothing without putting pressure on the body and was recorded to the closest 0.5 cm. Novel anthropometric judgment are new anthropometric indices that better assess a person's condition and include ABSI, AVI, BRI. BRI, in 2013, was defined as 364.2−365.5 {1−[(WC/2π/(0.50 height)^2^]} × 0.5. BRI predicts the visceral adipose tissue and percentage of body fat and ranges from 1 to 16 (Thomas et al., 2013). ABSI has been developed as a way to quantify the risk correlated with abdominal obesity, and defined as: $\frac{\mathrm{WC}}{{\mathrm{BMI}}^{2/3}\;{\text{height}}^{1/2}}$ (Krakauer & Krakauer, 2012). AVI is calculated using the formula: [2 cm (waist)^2^ + 0.7 cm (waist–hip)^2^]/1000, where both hip and waist measurements are measured in centimeters (Guerrero‐Romeroa & Rodrı'guez‐Mora, 2003).

### 2.5 Assessment of additional parameters

The left arm's systolic and diastolic blood pressure (SBP & DBP) were taken multiple times, ensuring a minimum rest period of 5 minutes in a calm, seated posture. The measurements were carried out by skilled technicians. The onset of distinct tapping sounds (Korotkoff phase 1) was used to define SBP, while the cessation of these sounds (Korotkoff phase 5) indicated DBP. We calculated the mean measurements of the two blood pressures and used them in the analysis. Numerous questions were asked about income, occupation, education, number of family members, travel abroad and inside the country, car ownership, home ownership, the number of rooms in the house and having modern furniture in the house to assess the socioeconomic status. A demographic questionnaire was also used to record demographic characteristics, and physical activity hours were multiplied by the corresponding metabolic equivalent task (MET‐h/week) (Asghari et al., 2012).

### 2.6 Statistical analysis

We checked the normality of our data through the Kolmogorov–Smirnov test. Since all the data were normal, we reported quantitative data as mean ± SD and qualitative data as percentage. SPSS 20.0 software (SPSS Inc) was used for statistical analysis of data. The quantity and quality of carbohydrates, proteins, and fats were presented as tertiles, where tertile 1 had the lowest values and tertile 3 had the highest values. Through logistic regression, we obtained the odds ratio and 95% CI, then reported each of them. The models in the table include model 1 and model 2 where model 1 is the crude model and model 2 is the adjusted model. The covariates included in our study were selected based on their potential to confound the relationship between our primary exposure (micronutrient intake) and outcomes (anthropometric indices, lipid accumulation, and blood lipid risk index). These covariates were chosen based on a review of the literature and our understanding of the biological relationships between these variables.

## 3 RESULTS

The demographic characteristics of 556 women aged 20–50 years across macronutrient quality tertiles are reported in Table 1 and Table S1. Aromatic amino acids branched chain amino acids (AAA/BCAA) were significantly associated with low socio‐economic status (SES) (*p* < .001). Participants in the highest tertile of glycemic load (GL), and AAA/ BCAA, had significantly lower age (*p* < .0001) compared to participants in the lowest tertile. In addition to low SES (*p* = .046), GL was significantly associated with low physical activity (*p* = .001). There was an association between weight (*p* = .007), height (*p* = .013), low physical activity (*p* < .0001), and MUFA/PUFA.

**TABLE 1:** Demographic characteristics of Tehranian women based on the quality of macronutrients.^a^

Variables	GL tertiles			*p* value^b^	AAA/BCAA tertiles			*p* value	MUFA/PUFA tertiles			*p* value
	T1	T2	T3		T1	T2	T3		T1	T2	T3	
	≤149.03	149.04–233.09	≥233.10		≤0.64	0.65–0.66	≥0.67		≤1.08	1.09–1.44	≥1.45	
	*n* = 191	*n* = 183	*n* = 186		*n* = 186	*n* = 187	*n* = 188		*n* = 186	*n* = 188	*n* = 187	
Age (years)	38.8 ± 8.1	34.5 ± 9.0	31.0 ± 6.6	<.0001	39.4 ± 7.9	34.0 ± 8.6	31.2 ± 7.2	<.0001	36.9 ± 9.0	32.1 ± 7.7	35.5 ± 8.3	.576
Weight (kg)	67 ± 11.6	65.5 ± 11.8	65.8 ± 12.1	.904	67.7 ± 12.1	65.8 ± 11.9	64.7 ± 11.4	.100	68.0 ± 13.2	66.0 ± 10.7	64.1 ± 11.2	.007
Height (cm)	163.7 ± 8.0	163.9 ± 7.2	163.3 ± 6.1	.143	164.5 ± 8.4	163.2 ± 6.5	163.4 ± 6.2	.075	163.3 ± 7.9	165.0 ± 6.1	162.7 ± 7.0	.013
BMI (kg/m^2^)	24.9 ± 3.9	24.3 ± 3.9	24.6 ± 4.3	.483	25.0 ± 4.2	24.6 ± 3.7	24.2 ± 4.2	.441	25.4 ± 4.3	24.3 ± 4.0	24.2 ± 3.8	.095
PA (MET‐min/week)												
Low (%)	104 (54.5)	86 (47.0)	71 (38.2)	.001	92 (49.87)	84 (44.9)	85 (45.2)	.670	99 (53.2)	69 (36.7)	93 (50.0)	<.0001
Medium (%)	61 (31.9)	53 (29.0)	58 (31.2)		57 (30.8)	55 (29.4)	60 (31.9)		57 (30.6)	53 (28.2)	62 (33.3)	
High (%)	26 (13.6)	44 (24.0)	57 (30.6)		36 (19.5)	48 (25.7)	43 (22.9)		30 (16.1)	66 (35.1)	31 (16.7)	
SES												
Low (%)	81 (42.4)	89 (48.6)	62 (33.3)	.046	85 (45.9)	89 (47.6)	58 (30.9)	<.0001	84 (45.2)	82 (43.6)	66 (35.5)	.229
Medium (%)	52 (27.2)	50 (27.3)	63 (33.9)		38 (20.5)	50 (26.7)	77 (41.0)		47 (25.3)	58 (30.9)	60 (32.3)	
High (%)	58 (30.4)	44 (24.0)	61 (32.8)		62 (33.5)	48 (25.7)	53 (28.2)		55 (29.6)	48 (25.5)	60 (32.3)	

Abbreviations: AAA, aromatic amino acid; BCAA, branched chain amino acid; BMI , body mass index; GL, glycemic load; MUFA, monounsaturated fatty acid; PA, physical activity; PUFA, polyunsaturated fatty acid; SES, socio‐economic status.

^a^ Data were presented as mean ± SD for continuous variables or *n* (%) for categorical variables.

^b^ *p*‐values are from one‐way analysis of variance (anova) for quantitative variables and Chi‐square test for qualitative variables.

Dietary intake across macronutrient quality tertiles is presented in Table 2 and Table S2. Participants in the highest tertile of GL had higher intake of energy, fiber, iron, magnesium, zinc, potassium, phosphorus, calcium, Vitamins A, D, E, K, C, B1, B3, B5, B6, B9, B2, B12, biotin, fruits, vegetables, grains, rice, pasta, legumes, potatoes, meat, poultry, eggs, dairy, oils, and fats (*p* < .05), compared to the lowest tertile. Higher intakes of vitamin A and B1 and lower intakes of vitamin E, cereals and rice, pasta, legumes, potatoes were observed in the highest tertile of MUFA/PUFA. Relationship between higher tertile of AAA/BCAA and higher intake of energy, fiber, iron, magnesium, zinc, phosphorus, vitamins A, K, C, B1, B3, B5, B6, B9, B2, vegetables, rice, pasta, legumes, potatoes, meat, poultry, eggs, and oils and fats was observed, while people who were in the highest tertile of AAA/BCAA had lower intake of calcium, fruits, and dairy products.

**TABLE 2:** Tehranian women's dietary intake based on the quality of macronutrients.^a^

Variables	GL tertiles			*p* value^b^	AAA/BCAA tertiles			*p* value	MUFA/PUFA tertiles			*p* value
	T1	T2	T3		T1	T2	T3		T1	T2	T3	
	≤149.03	149.04–233.09	≥233.10		≤0.64	0.65–0.66	≥0.67		≤1.08	1.09–1.44	≥1.45	
	*n* = 191	*n* = 183	*n* = 186		*n* = 186	*n* = 187	*n* = 188		*n* = 186	*n* = 188	*n* = 187	
Energy (kcal/day)	1397.5 ± 416	2124.5 ± 428.5	3008.4 ± 629.2	<.0001	1809.7 ± 747.0	2284.4 ± 816.8	2409.1 ± 802.8	<.0001	1949.1 ± 793.2	2419.8 ± 820.0	2134.9 ± 809.9	.295
Fiber (g/day)	3.7 ± 2.0	5.6 ± 2.5	7.3 ± 2.7	<.0001	4.7 ± 2.6	6.1 ± 3.1	5.8 ± 2.5	<.0001	4.6 ± 2.6	6.5 ± 3.1	5.5 ± 2.5	.369
Iron (mg/day)	11.9 ± 4.7	17.1 ± 4.9	22.6 ± 6.0	<.0001	14.0 ± 5.8	17.4 ± 6.0	20.0 ± 7.2	<.0001	16.0 ± 6.8	18.5 ± 7.0	17.0 ± 6.4	.151
Magnesium (mg/day)	164.7 ± 73.4	248.4 ± 71.3	332.1 ± 96.8	<.0001	212.0 ± 100.1	274.5 ± 117.6	256.0 ± 90.1	.001	221.8 ± 99.4	275.2 ± 107.5	245.4 ± 105.5	.858
Zinc (mg/day)	5.8 ± 3.5	8.3 ± 2.7	11.1 ± 3.9	<.0001	6.9 ± 3.5	9.5 ± 4.6	8.7 ± 3.5	<0.0001	7.2 ± 3.8	9.5 ± 3.9	8.4 ± 4.1	.396
Potassium (mg/day)	2032.1 ± 914.0	2947.0 ± 1022.0	3982.2 ± 1281.1	<.0001	2664.4 ± 1310.3	3275.6 ± 1512.7	2991.1 ± 1117.2	.094	2641.7 ± 1314.8	3317.7 ± 1375.7	2969.8 ± 1259.5	.639
Phosphorus (mg/day)	720.7 ± 363.4	1142.9 ± 370.0	1574.4 ± 513.2	<.0001	979.9 ± 513.0	1237.3 ± 579.1	1207.2 ± 511.9	.001	1002.4 ± 519.5	1302.2 ± 516.9	1119.4 ± 563.2	.722
Calcium (mg/day)	625.1 ± 289.6	916.0 ± 305.5	1214.2 ± 425.5	<.0001	952.7 ± 463.7	947.0 ± 459.7	849.4 ± 317.2	.002	811.9 ± 361.7	1007.8 ± 401.2	927.8 ± 470.8	.710
Vitamin A (RAE/day)	762.7 ± 675.8	1066.4 ± 691.0	1598.7 ± 923.0	<.0001	946.0 ± 789.6	1266.2 ± 950.1	1204.9 ± 747.9	<.0001	768.0 ± 596.2	1411.5 ± 991.6	1235.6 ± 760.9	.001
Vitamin D (μg/day)	0.7 ± 1.1	1.1 ± 1.2	1.9 ± 1.5	<.0001	1.1 ± 1.4	1.3 ± 1.4	1.4 ± 1.2	.725	0.9 ± 1.2	1.6 ± 1.6	1.2 ± 1.2	.842
Vitamin E (mg/day)	10.0 ± 5.9	12.6 ± 7.2	15.2 ± 5.9	<.0001	11.9 ± 5.5	12.3 ± 7.7	13.5 ± 6.6	.258	16.2 ± 7.2	13.4 ± 5.3	8.1 ± 4.6	<.0001
Vitamin K (μg/day)	95.6 ± 70.7	128.5 ± 69.7	165.8 ± 89.4	<.0001	109.2 ± 60.3	139.4 ± 90.2	139.9 ± 88.9	<.0001	104.3 ± 53.7	148.8 ± 100.7	135.3 ± 78.8	.959
Vitamin C (mg/day)	89.8 ± 52.1	113.5 ± 58.4	170.4 ± 103.6	<.0001	111.0 ± 81.4	133.4 ± 90.1	128.0 ± 72.5	.040	105.9 ± 82.1	139.2 ± 91.0	127.2 ± 68.3	.182
Vitamin B1 (mg/day)	1.1 ± 0.4	1.8 ± 0.4	2.8 ± 0.8	<.0001	1.5 ± 0.7	2.0 ± 0.8	2.2 ± 0.9	<.0001	1.7 ± 0.7	2.1 ± 0.9	1.8 ± 0.8	.035
Vitamin B3 (mg/day)	14.5 ± 6.8	21.2 ± 6.4	31.0 ± 9.2	<.0001	17.0 ± 7.5	23.5 ± 10.3	26.0 ± 10.2	<.0001	19.5 ± 9.8	25.2 ± 10.5	21.8 ± 9.4	.787
Vitamin B5 (mg/day)	3.3 ± 1.7	5.0 ± 1.7	7.0 ± 2.2	<.0001	4.4 ± 2.2	5.5 ± 2.6	5.3 ± 2.2	.008	4.6 ± 2.5	5.7 ± 2.3	4.9 ± 2.3	.287
Vitamin B6 (mg/day)	1 ± 0.5	1.5 ± 0.5	2.3 ± 0.8	<.0001	1.2 ± 0.7	1.7 ± 0.9	1.8 ± 0.8	<.0001	1.4 ± 0.8	1.8 ± 0.9	1.5 ± 0.7	.622
Vitamin B9 (μg/day)	198.5 ± 111.8	284.4 ± 118.1	399.8 ± 169.0	<.0001	235.7 ± 104.3	320.5 ± 186.8	323.1 ± 157.1	<.0001	266.2 ± 150.6	338.9 ± 183.4	274.3 ± 126.2	.326
Vitamin B2 (mg/day)	1.1 ± 0.6	1.7 ± 0.6	2.5 ± 0.8	<.0001	1.6 ± 0.8	1.9 ± 1.0	1.9 ± 0.8	.027	1.5 ± 0.7	2.1 ± 0.9	1.8 ± 0.9	.890
Vitamin B12 (μg/day)	2.1 ± 1.6	3.2 ± 1.6	4.5 ± 2.2	<.0001	2.8 ± 1.7	3.7 ± 2.3	3.3 ± 2.0	.073	2.5 ± 1.9	3.9 ± 1.9	3.3 ± 2.1	.180
Biotin (μg/day)	8.3 ± 5.5	13.8 ± 6.1	22.3 ± 8.9	<.0001	12.3 ± 8.5	16.2 ± 10.3	15.5 ± 7.6	.078	12.8 ± 9.8	17.0 ± 8.0	14.3 ± 8.8	.530
Fruits (g/day)	206.4 ± 117.2	268.9 ± 207.4	334.7 ± 256.7	<.0001	270.1 ± 203.5	311.9 ± 251.0	225.6 ± 148.8	.021	261.2 ± 186.7	274.0 ± 227.5	272.1 ± 208.3	.838
Vegetables (g/day)	235.6 ± 171.5	332.3 ± 213.4	394.1 ± 229.7	<.0001	287.6 ± 183.8	343.8 ± 231.9	327.5 ± 225.2	.023	264.6 ± 172.4	376.1 ± 268.6	317.8 ± 178.0	.573
Grains (g/day)	320.3 ± 94.6	470.1 ± 120.7	722.0 ± 219.9	<.0001	509.5 ± 193.1	535.0 ± 249.4	611.8 ± 227.6	.074	591.7 ± 240.6	547.2 ± 237.3	564.4 ± 224.4	.045
Rice, pasta, legumes, potatoes (g/day)	170.8 ± 99.3	281.4 ± 114.4	406.7 ± 206.3	<.0001	216.3 ± 148.2	321.5 ± 197.5	316.9 ± 159.4	<.0001	289.6 ± 199.4	288.3 ± 141.9	277.5 ± 183.4	.003
Meat, poultry, eggs (g/day)	53.2 ± 59.6	65.0 ± 48.5	94.8 ± 73.9	<.0001	43.4 ± 39.1	90.2 ± 76.8	78.6 ± 60.6	<.0001	47.3 ± 42.0	84.7 ± 71.6	80.2 ± 67.4	.094
Dairy (g/day)	228.8 ± 163.9	350.4 ± 198.2	429.0 ± 267.1	<.0001	397.7 ± 241.7	343.7 ± 261.6	267.3 ± 151.9	<.0001	317.6 ± 210.9	360.0 ± 232.6	329.4 ± 242.4	.532
Oils and fats (g/day)	19.9 ± 10.2	27.2 ± 19.5	37.2 ± 21.0	<.0001	24.5 ± 18.4	30.6 ± 20.2	28.9	.010	24.1 ± 14.1	31.4 ± 19.9	28.5 ± 21.2	.201

Abbreviations: AAA, aromatic amino acid; BCAA, branched chain amino acid; GL, glycemic load; MUFA, monounsaturated fatty acid; PUFA, polyunsaturated fatty acid.

^a^ Data were presented as mean ± SD.

^b^ *p*‐values are from one‐way analysis of variance (anova).

The association between the participants' anthropometric and lipid indices based on the tertile of gram of macronutrients quantity were described in Table S3. In terms of macronutrient percentage in Table 3, participants in the highest tertile of protein intake had significantly lower odds of obesity (OR: 0.57, 95% CI: 0.37–0.90; *p* = .012) and lower odds of LAP (OR: 0.57, 95% CI: 0.35–0.93; *p* = .011) compared to participants in the lowest tertile.

**TABLE 3:** The association between Tehranian women's anthropometric and lipid indices based on the macronutrients amount (%).^a^

Variables	Carbohydrate tertiles (%)			*p* trend^b^	Protein tertiles (%)			*p* trend	Fat tertiles (%)			*p* trend
	T1	T2	T3		T1	T2	T3		T1	T2	T3	
	≤57.61	57.62–63.79	≥63.80		≤12.92	12.93–14.97	≥14.98		≤24.28	24.29–29.55	≥29.56	
	*n* = 184	*n* = 188	*n* = 188		*n* = 186	*n* = 191	*n* = 183		*n* = 186	*n* = 186	*n* = 188	
Obesity												
Model 1	1	0.86 (0.57–1.30)	1.27 (0.85–1.92)	.202	1	0.70 (0.47–1.06)	0.50 (0.33–0.77)	.001	1	0.73 (0.49–1.11)	0.88 (0.59–1.32)	.494
Model 2	1	0.78 (0.50–1.22)	0.98 (0.63–1.54)	.994	1	0.64 (0.42–0.98)	0.57 (0.37–0.90)	.012	1	0.95 (0.60–1.50)	1.22 (0.78–1.92)	.395
New anthropometric indices												
BRI												
Model 1	1	0.84 (0.56–1.28)	1.38 (0.90–2.13)	.117	1	0.74 (0.48–1.14)	0.58 (0.38–0.89)	.013	1	0.57 (0.37–0.87)	0.76 (0.49–1.17)	.181
Model 2	1	0.84 (0.54–1.33)	0.96 (0.60–1.56)	.897	1	0.79 (0.50–1.25)	0.75 (0.48–1.20)	.229	1	0.79 (0.49–1.27)	1.01 (0.62–1.64)	.968
LAP												
Model 1	1	0.91 (0.59–1.39)	0.87 (0.57–1.34)	.536	1	0.53 (0.34–0.80)	0.45 (0.29–0.70)	<.0001	1	0.85 (0.56–1.30)	0.60 (0.39–0.93)	.025
Model 2	1	0.97 (0.53–1.42)	1.43 (0.88–2.32)	.113	1	0.45 (0.28–0.72)	0.57 (0.35–0.93)	.011	1	1.05 (0.64–1.70)	0.77 (0.47–1.26)	.316
Blood lipid risk index												
CRI‐1												
Model 1	1	0.94 (0.62–1.44)	1.13 (0.74–1.72)	.530	1	0.78 (0.51–1.19)	1.03 (0.68–1.56)	.946	1	0.57 (0.37–0.87)	1.05 (0.70–1.58)	.944
Model 2	1	1.04 (0.65–1.66)	1.15 (0.71–1.86)	.568	1	0.77 (0.49–1.22)	1.36 (0.85–2.18)	.260	1	0.53 (0.32–0.87)	0.88 (0.55–1.42)	.568
AC												
Model1	1	0.65 (0.40–1.04)	0.88 (0.54–1.44)	.715	1	0.94 (0.58–1.53)	0.75 (0.47–1.21)	.244	1	0.71 (0.44–1.14)	1.01 (0.62–1.65)	.972
Model2	1	0.58 (0.35–0.99)	0.97 (0.56–1.68)	.922	1	0.93 (0.56–1.56)	0.77 (0.46–1.29)	.326	1	0.55 (0.32–0.94)	0.80 (0.46–1.37)	.369
AIP												
Model 1	1	0.73 (0.44–1.22)	1.44 (0.81–2.55)	.185	1	0.65 (0.38–1.13)	0.71 (0.41–1.24)	.227	1	0.44 (0.25–0.77)	0.74 (0.41–1.33)	.288
Model 2	1	0.67 (0.38–1.17)	1.15 (0.61–2.17)	.672	1	0.66 (0.37–1.18)	0.98 (0.54–1.78)	.921	1	0.48 (0.26–0.88)	0.88 (0.46–1.68)	.726

*Note*: Obesity: BMI ≥ 30 g/m^2^. Model 1: crude. Model 2: adjusted for age (year), marital status, physical activity (MET/h), Socio‐economic status, total energy intake (kcal).

Abbreviations: AC, atherogenic coefficient (for men >5 and for women >4.5); AIP, atherogenic index of plasma (low risk: AIP <0.11, intermediate risk: AIP , 0.11–0.21, high risk: AIP > 0.21); BRI, body roundness index (narrow body: 1–20, round body >20); CRI‐1, Castelli's risk index 1 (Castelli risk index‐I (TC/HDL‐C) ≥5.0); LAP, lipid accumulation product.

^a^ Data are presented as OR (95% CI).

^b^ Obtained from binary logistic regression.

Table 4 outlines the relationship between the anthropometric and lipid indices of the participants based on the quality of carbohydrate intake. After adjusting for potential confounding variables, it was found that participants in the highest glycemic index (GI) and glycemic load (GL) tertiles were more likely to have abdominal circumference (AC) compared to those in the lowest tertile, with odds ratios (OR) of 2.53 (95% CI: 1.45–4.39; *p* = .001) and 3.67 (95% CI: 1.42–9.48; *p* = .004), respectively. Participants in the highest GL tertile were more likely to be obese (OR: 2.18, 95% CI: 1.05–4.52; *p* = .015) and less likely to have lipid accumulation product (LAP) (OR: 0.46, 95% CI: 0.25–0.83; *p* = .009) compared to those in the lowest tertile in the adjusted model. However, no significant association was observed between GI or GL and the anthropometric and lipid indices (*p* ≥ .05).

**TABLE 4:** The association between Tehranian women's anthropometric and lipid indices based on the carbohydrates quality.^a^

Variables	GI tertiles			*p* trend^b^	GL tertiles			*p* trend
	T1	T2	T3		T1	T2	T3	
	≤53.84	53.85–62.79	62.80+		≤149.03	149.04–233.09	233.10+	
	*n* = 186	*n* = 188	*n* = 186		*n* = 191	*n* = 183	*n* = 186	
Obesity								
Model 1	1	0.96 (0.64–1.44)	0.67 (0.44–1.01)	.057	1	0.58 (0.38–0.88)	0.81 (0.54–1.22)	.460
Model 2	1	1.26 (0.81–1.95)	1.00 (0.63–1.59)	.980	1	0.89 (0.53–1.49)	2.18 (1.05–4.52)	.015
New anthropometric indices								
BRI								
Model 1	1	0.90 (0.58–1.39)	0.64 (0.42–0.98)	.039	1	0.67 (0.43–1.03)	0.61 (0.40–0.94)	.034
Model 2	1	1.49 (0.94–2.37)	1.50 (0.93–2.41)	.106	1	1.00 (0.59–1.69)	1.41 (0.68–2.95)	.715
LAP								
Model 1	1	0.61 (0.40–0.94)	0.69 (0.45–1.05)	.087	1	0.76 (0.49–1.17)	0.88 (0.58–1.35)	.652
Model 2	1	0.88 (0.55–1.42)	1.23 (0.75–2.01)	.408	1	0.36 (0.16–0.80)	0.46 (0.25–0.83)	.009
Blood lipid risk index								
CRI‐1								
Model 1	1	0.84 (0.55–1.27)	0.73 (0.48–1.10)	.136	1	0.75 (0.50–1.14)	0.58 (0.38–0.88)	.012
Model 2	1	1.07 (0.67–1.71)	1.09 (0.68–1.76)	.718	1	1.13 (0.67–1.90)	1.45 (0.68–3.06)	.323
AC								
Model 1	1	1.16 (0.73–1.84)	1.62 (1.00–2.62)	.052	1	0.70 (0.44–1.11)	1.38 (0.84–2.27)	.144
Model 2	1	1.68 (1.00–2.83)	2.53 (1.45–4.39)	.001	1	1.07 (0.59–1.94)	3.67 (1.42–9.48)	.004
AIP								
Model 1	1	0.57 (0.32–1.0)	0.64 (0.36–1.12)	.146	1	0.44 (0.25–0.78)	0.50 (0.28–0.89)	.041
Model 2	1	0.83 (0.46–1.50)	1.10 (0.60–2.03)	.672	1	0.66 (0.33–1.30)	1.10 (0.42–2.85)	.596

*Note*: Obesity: BMI ≥ 30 g/m^2^. Model 1: crude. Model 2: adjusted for age (year), marital status, physical activity (MET/h), Socio‐economic status, total energy intake (kcal).

Abbreviations: AC, atherogenic coefficient (for men >5 and for women >4.5); AIP, atherogenic index of plasma (low risk: AIP <0.11, intermediate risk: AIP = 0.11–0.21, high risk: AIP > 0.21); BRI, body roundness index (narrow body: 1–20, round body >20); CRI‐1, Castelli's risk index 1 (Castelli risk index‐I (TC/HDL‐C) ≥5.0); GI, glycemic index; GL, glycemic load; LAP, Lipid accumulation product.

^a^ Data are presented as OR (95% CI).

^b^ Obtained from binary logistic regression.

The association between Tehranian women's anthropometric and lipid indices based on quality of proteins and fats were described in Table 5. Participants in the highest tertile of AAA/BCAAs had lower CRI‐1 (OR: 0.36, 95% CI: 0.21–0.6; *p* < .0001) and AIP (OR: 0.19, 95% CI: 0.08–0.42; *p* <.0001) in comparison with the lowest tertile. Obesity (OR: 0.65, 95% CI: 0.42–0.99; *p* = .041) and AIP (OR: 0.40, 95% CI: 0.21–0.75; *p* = .004) in the highest tertile of MUFA/PUFA were lower than the lowest one. No significant associations were found between the quality of proteins and fats with other anthropometric and lipid indices (*p* ≥ .05).

**TABLE 5:** The association between Tehranian women's anthropometric and lipid indices based on the quality of proteins and fats^a^.

Variables	AAA/BCAA tertiles (g)			*p* trend^b^	MUFA/PUFA tertiles (g)			*p* trend	SAF/PUFA tertiles (g)			*p* trend
	T1	T2	T3		T1	T2	T3		T1	T2	T3	
	≤0.64	0.65–0.66	≥0.67		≤1.08	1.09–1.44	≥1.45		≤1.00	1.01–1.37	≥1.38	
	*n* = 186	*n* = 187	*n* = 188		*n* = 186	*n* = 188	*n* = 187		*n* = 186	*n* = 188	*n* = 187	
Obesity												
Model 1	1	0.62 (0.41–0.93)	0.69 (0.46–1.03)	.059	1	0.49 (0.32–0.74)	0.61 (0.40–0.91)	.011	1	0.47 (0.31–0.71)	0.56 (0.37–0.84)	.018
Model 2	1	0.80 (0.51–1.25)	1.11 (0.69–1.80)	.692	1	0.66 (0.42–1.04)	0.65 (0.42–0.99)	.041	1	0.57 (0.36–0.89)	0.64 (0.41–0.99)	.096
New anthropometric indices												
BRI												
Model 1	1	0.70 (0.45–1.08)	0.83 (0.54–1.27)	.002	1	0.52 (0.34–0.80)	0.61 (0.39–0.95)	.024	1	0.60 (0.39–0.92)	0.57 (0.37–0.87)	.024
Model 2	1	1.25 (0.76–2.03)	0.70 (0.43–1.15)	.134	1	0.61 (0.38–0.98)	0.66 (0.42–1.05)	.076	1	0.68 (0.42–1.04)	0.69 1 (0.43–1.09)	.187
LAP												
Model 1	1	0.32 (0.21–0.51)	0.53 (0.34–0.80)	.001	1	0.83 (0.54–1.28)	0.77 (0.50–1.18)	.221	1	0.53 (0.34–.82)	0.60 (0.39–0.91)	.039
Model 2	1	0.44 (0.26–0.72)	1.08 (0.65–1.81)	.977	1	1.15 (0.70–1.88)	0.86 (0.54–1.38)	.566	1	0.58 (0.35–0.94)	0.66 (0.41–1.07)	.156
Blood lipid risk index												
CRI‐1												
Model 1	1	0.50 (0.33–0.75)	0.26 (0.17–0.40)	<.0001	1	1.00 (0.66–1.51)	0.69 (0.45–1.05)	.099	1	0.82 (0.54–1.24)	0.98 (0.64–1.48)	.953
Model 2	1	0.61 (0.38–1.0)	0.36 (0.21–0.60)	<.0001	1	1.16 (0.72–1.87)	0.76 (0.48–1.20)	.270	1	0.95 (0.59–1.54)	1.12 (0.71–1.78)	.574
AIP												
Model 1	1	0.67 (0.41–1.10)	0.56 (0.34–0.91)	.019	1	0.46 (0.26–0.84)	0.39 (0.22–0.70)	.002	1	0.49 (0.27–0.86)	0.50 (0.28–0.88)	.041
Model 2	1	0.23 (0.10–0.52)	0.19 (0.08–0.42)	<.0001	1	0.56 (0.29–1.05)	0.40 (0.21–0.75)	.004	1	0.53 (0.28–1.00)	0.60 (0.32–1.10)	.213

*Note*: Obesity: BMI ≥ 30 g/m^2^. Model 1: crude. Model 2: adjusted for age (year), marital status, physical activity (MET/h), socio‐economic status, total energy intake (kcal).

Abbreviations: AAA, aromatic amino acid; AC, atherogenic coefficient (for men >5 and for women >4.5); AIP, atherogenic index of plasma (low risk: AIP <0.11, intermediate risk: AIP = 0.11–0.21, high risk: AIP > 0.21); BCAA, branched chain amino acid; BRI, body roundness index (narrow body: 1–20, round body >20); CRI‐1, Castelli's risk index 1 (Castelli risk index‐I (TC/HDL‐C) ≥5.0); LAP, lipid accumulation product; MUFA, monounsaturated fatty acid; PUFA, polyunsaturated fatty acid; SFA, saturated fatty acid.

^a^ Data are presented as OR (95% CI).

^b^ Obtained from binary logistic regression.

## 4 DISCUSSION

In the current study, there was a significant relationship between quantity of carbohydrate intake and obesity. In addition, the percentage of protein received was associated with obesity and LAP. The relationship between the gram of fat intake and CRI‐1 (*p* < .001) was also observed. GI was associated with AC, and GL was associated with obesity, LAP, and AC. AAA to BCAA ratio indices were associated with CRI‐1 and AIP, and MUFA to PUFA ratio was related to obesity and AIP. In the multivariable‐adjusted model, no correlation was found between the quality and quantity of macronutrients and other indicators. This study is the first of its kind to investigate the link between the quality and quantity of dietary macronutrient indices and factors such as obesity, novel anthropometric indices, lipid accumulation, and the risk index for blood lipids.

The prevalence of obesity has increased in the last three decades (Shabana et al., 2020). In terms of carbohydrate percentage, contrary to our study, a systematic review and meta‐analysis showed that higher percentage of carbohydrate intakes could not increase the likelihood of obesity (Sartorius et al., 2018). In this meta‐analysis, there was a lot of heterogeneity in the included studies.

In the present study, the participants in the highest tertile of protein percentage had 47% lower odds of having obesity and LAP, compared to the lowest tertile. No previous study has been done on the field of protein intake and LAP, so we used articles that examined the components of LAP (WC and serum TG) (Ahn et al., 2019). In agreement with our finding, Simonson et al. found that different proteins, both in quality and quantity, can be considered as a treatment for the parameters of the metabolic syndrome and appetite regulation in obese patients (Simonson et al., 2020). Also, after energy adjustment, Baba et al. showed more weight loss on a high‐protein diet (Baba et al., 1999). Similarly, in a representative sample of Koreans ≤60 years of age, total protein intake was revealed to be negatively associated with BMI and WC (Park et al., 2018). In addition, Clifton & Keogh (2007) found that high‐protein, low‐carbohydrate diets reduced TG levels, especially in people with high TG levels. Among the opposing studies was the study of Witjaksono et al. indicated that differences in protein ratio, regardless of protein composition in the diet, did not influence WC among adults with central obesity (Witjaksono et al., 2018). The Witjaksono et al.'s study had several limitations: First, it was an open‐randomized clinical trial in which the researcher and individuals knew about the intervention. Second, its participants had low compliance. Third, the possibility of potential selection bias due to the significant number of participants who were lost during the measurement of initial outcomes, and there was also a recall bias because some contributors did not fill their own log‐book every day. Halkjær et al. (2011) also observed that high protein intake was not associated with weight loss or increased WC, where in this study, weight and WC were reported spontaneously during follow‐up at four of the six study centers, potentially increasing heterogeneity between centers.

In terms of macronutrient quality, in our study, people in the highest GI tertile had the highest AC, and individuals in the highest GL tertile had the highest levels of obesity, AC, and the lowest LAP level. In agreement with this result was Liese et al. (2005) study, which indicated no association between GI and BMI after adjusting for demographic characteristics, energy expenditure, smoking, and family history of diabetes. A study on the relationship between GI and AC has not been done before, but since AC is equal to non‐HDL/HDL, our results were in agreement with former cohort studies among adults, because they have demonstrated that GI scores are inversely related to HDL‐c (Hosseinpour‐Niazi et al., 2013; Salmeron et al., 1997; Salmerón et al., 1997). Similarly, two separate cross‐sectional studies have indicated that diets with a low glycemic index (GI) are linked to elevated levels of HDL‐c, particularly in women (Ford & Liu, 2001; Frost et al., 1999). There is no study on the relationship between GL and AC or LAP, but many studies have identified a link between diets high in GI and risk factors of CVD such as high TG and low HDL‐c (Hang et al., 2016), which were in line with our results. Murakami et al. also showed that dietary GL is independently negatively associated with HDL‐c (Murakami et al., 2006), which, according to the AC formula, was in line with our results. The literature indicated that refined carbohydrates and sugar cause obesity, while unrefined carbohydrates might have the opposite effect (Aller et al., 2011; Jebb, 2005; Jebb, 2014). Since refined grains have a high glycemic load (Gangwisch et al., 2015), the results of these studies were also similar to the result of the present study. Contrary to our study, we can mention the study of Kim et al. (2018) in which the carbohydrates quality consumed was negatively related to the prevalence of obesity, and for carbohydrate quality used the GI index. Conversely, in our research, we found no association between the glycemic index (GI) and obesity. The dietary assessment tool used for food evaluation in Kim et al.'s study was 24‐h recall, which was different from our study.

For fat quality, we used two indicators, SFA to PUFA and MUFA to PUFA ratio. Obesity and AIP were lower in the highest MUFA/PUFA category than in the lowest category. These findings are consistent with an ecological study by Moussavi et al. which showed that populations with a lower obesity prevalence seemed to consume more MUFA (Moussavi et al., 2008a). However, a review study has found inconclusive results on the relationship between obesity and the fatty acid type consumed, and found that although animal studies mention a possible link between obesity and the fatty acid type consumed, this issue seems complex (Moussavi et al., 2008b).

In the present study, people in the highest GI category had the highest AC, which could be due to a decrease in HDL. Carbohydrate‐drink meals, which cause high post‐prandial hyperglycemic reactions in adults, increase urinary chromium excretion (Anderson et al., 1990). Chromium depletion has been demonstrated to be associated with dyslipidemia and hyperglycemia (Anderson, 1999). Furthermore, following a meal with a high glycemic index (GI), there is a surge in both glucose and insulin levels. However, after a span of 4–6 h, glucose levels may drop to hypoglycemic ranges. This triggers the release of counter‐regulatory hormones, leading to an increase in both glucose and free fatty acid concentrations (Ludwig, 2002). The elevated levels of glucose, free fatty acids, and insulin can induce insulin resistance (Boden et al., 1994; Del Prato et al., 1994; Ludwig, 2002; Rossetti et al., 1990), which in turn can lead to an increase in triglycerides (TG) and a decrease in HDL cholesterol. (Reaven, 1995).

In the current study, the increased AC following the increment of GL could be due to a decrease in HDL and an increase in TC. Increased glycemic load is assumed to cause a pro‐atherogenic profile by increasing triglycerides and small dense LDL‐concentrations. Also, HDL‐c concentration is decreased by the production of fatty acids in the liver, while lipoprotein lipase function is blocked through increased production of apolipoprotein CIII, especially in the presence of insulin resistance (Grundy, 1998; Grundy et al., 2002; Liu & Willett, 2002).

In this research, it was found that a higher proportion of energy intake from proteins was linked to a reduced likelihood of obesity. There are several proposed mechanisms that could explain the observed relationship between protein intake and obesity. Firstly, protein provides the least amount of energy among macronutrients and requires more energy for metabolism compared to fats or carbohydrates (Feinman & Fine, 2003; Fine & Feinman, 2004). Secondly, protein contributes to feelings of fullness, which can lead to a reduction in food intake. (Halton & Hu, 2004). Protein intake has been found to stimulate ghrelin, a hormone that increases appetite, and to decrease the peptide YY, a hormone that suppresses appetite. These are the suggested underlying mechanisms for satiety (Batterham et al., 2006; Lee et al., 2009). Furthermore, cholecystokinin, which is released from the duodenum upon protein intake, is thought to suppress appetite in a similar manner (Verdich et al., 2001). Additionally, the secretion of glucagon‐like peptide 1 (GLP‐1) from the distal L cell of the small intestine, which is induced by protein consumption, slows the rate of gastric emptying and increases feelings of fullness (Kissileff et al., 1981). Thirdly, adequate protein intake in older individuals can enhance lean body mass and prevent sarcopenia, thereby increasing the basal metabolic rate and physical activity, and reducing the risk of obesity (Stenholm et al., 2008).

In the current study, the relationship between increasing protein intake and decreasing LAP was also detected. When there is an increase in energy intake from protein consumption, the percentage of energy intake from carbohydrates decreases. Since carbohydrates consumption causes an increase in triglyceride levels, the LAP index in the current study decreased by increasing the percentage of energy received from proteins.

It was shown that participants in the highest tertile of MUFA to PUFA ratio had the highest rate of obesity compared to participants in the lowest one. MUFA ≥ PUFA> SFA is the preferred sequence for oxidation in the body (Krishnan & Cooper, 2014). MUFA consumption increases the diet thermogenesis, which in turn stimulates the sympathetic nervous system. Also, people with abdominal obesity might be more responsive to the sympathetic nervous system stimulation because they have an enhanced sensibility of beta‐adrenoceptors. Fat oxidation and oxidative response to high‐fat meals or various diets with different fatty acid (FA) combinations may be influenced by sex or BMI status of participants. Overweight and obese women responded with a higher diet‐induced thermogenesis (DIT) and fat oxidation following a diet with high MUFA, compared to diet with high SFA while men did not (Bouchard et al., 1993).

The inverse relationship between CRI‐1 (Castelli's risk index 1) and the intake of fat in grams, as well as AAA/BCAA indices, could be attributed to several mechanisms. The negative correlation between CRI‐1 and fat intake could be due to metabolic rate variations. Variants of the cry 1 gene may influence the impact of fat intake on the resting metabolic rate in women who are overweight or obese (Mirzababaei et al., 2021). This suggests that genetic factors could play a role in how fat intake influences CRI‐1. Also, high‐fat dairy consumption has been found to be inversely associated with measures of adiposity and metabolic health (Kratz et al., 2013), which could potentially explain the inverse association between fat intake and CRI‐1, as high‐fat dairy products are a major source of dietary fat. As for the inverse association between CRI‐1 and AAA/BCAA (aromatic amino acids/branched chain amino acids) indices, one of the mechanisms which could be considered is blood pressure where increased levels of branched‐chain amino acids (BCAAs, i.e., leucine, isoleucine, and valine) have been found to be associated with higher blood pressure and risk of hypertension (Lin et al., 2022), which could potentially influence CRI‐1, which is a measure of cardiovascular risk. Also, alterations in BCAA metabolism have been found in a number of disease states, including obesity and type 2 diabetes (Holeček, 2018). These alterations could potentially influence CRI‐1.

While the present study has the strength to be the first to compare the quantity and quality of macronutrients with obesity, new anthropometric indices, lipid accumulation, and blood lipid risk index, there are some limitations. Our study has a cross‐sectional design, which makes it impossible to find out the causality. We also used the FFQ questionnaire to evaluate food intake, which depends on the person's memory. Gender restriction of the study population is another limitation of this study, which reduces generalizability.

In conclusion, obesity was positively associated with the quantity of carbohydrate intake and GL, while inversely associated with the percentage of protein intake, and the ratio of MUFA to PUFA. CRI‐1 was inversely associated with grams of fat intake and AAA/BCAA indices. LAP was inversely associated with dietary GL. AC was positively associated with GI and GL of carbohydrates, while AIP was inversely associated with AAA/BCAA and MUFA/PUFA indicators. Accordingly, this study adds to the cumulating evidence that a diet high in complex carbohydrate (low to medium glycemic index and glycemic load), lean protein (meats and legumes), and healthy fats (nuts and oils) could play an important role in decreasing obesity risk and contributing to overall cardiovascular health. However, future prospective studies are needed to confirm these findings.

## AUTHOR CONTRIBUTIONS

**Zahra Salehi:** Formal analysis (equal); investigation (equal); software (equal); writing – original draft (equal); writing – review and editing (equal). **Pegah Rahbarinejad:** Formal analysis (equal); investigation (equal); methodology (equal); software (equal); writing – review and editing (equal). **Batoul Ghosn:** Investigation (equal); visualization (equal); writing – review and editing (equal). **Leila Azadbakht:** Conceptualization (equal); data curation (equal); formal analysis (equal); investigation (equal); methodology (equal); project administration (equal); resources (equal); software (equal); supervision (equal); visualization (equal); writing – review and editing (equal).

## FUNDING INFORMATION

Research reported in this publication was supported by Elite Researcher Grant Committee under award number (995396) from the National Institute for Medical Research Development (NIMAD), Tehran, Iran.

## CONFLICT OF INTEREST STATEMENT

The authors declare that they do not have any conflict of interest.

## ETHICS STATEMENT

This study was approved by the institutional review board of the Tehran University of Medical Sciences.

## INFORMED CONSENT

Written informed consent was obtained from all study participants.

## Supporting information


Tables S1–S3.

## Data Availability

The data are not publicly available due to privacy or ethical restrictions.
