# Supplementary material for: Association of quality and quantity of macronutrients intake with obesity, new anthropometric indices, lipid accumulation, and blood lipid risk index in Tehranian women
Source: Food Sci Nutr. 2024 Feb 4;12(5):3237–50. doi: 10.1002/fsn3.3991 (PMC11077202; doi:10.1002/fsn3.3991)
Supplement: Supplementary file 1 — Tables S1–S3. [file FSN3-12-3237-s001.docx]

**Supplementary table 1.** Demographic characteristics of Tehranian women based on the quality of macronutrients.*

| variables | GI tertiles | | | P value** | SFA/ PUFA tertiles | | | P value |
| --- | --- | --- | --- | --- | --- | --- | --- | --- |
|  | T1 | T2 | T3 |  | T1 | T2 | T3 |  |
|  | ≤53.84 | 53.85 - 62.79 | ≥62.80 |  | ≤ 1.00 | 1.01- 1.37 | ≥1.38 |  |
|  | n=186 | n=188 | n=186 |  | n=186 | n=188 | n=187 |  |
| Age (year) | 38.5±8.5 | 34.0±8.3 | 32.0±7.6 | < 0.0001 | 37.2±8.9 | 33.4±8.1 | 34.0±8.3 | 0.428 |
| Weight (kg) | 68.2±12.5 | 65.2±11.0 | 64.9±11.8 | 0.616 | 68.5±13.0 | 65.0±9.8 | 64.7±12.1 | 0.002 |
| Height (cm) | 164.5±8.5 | 162.5±6.1 | 164.1±6.5 | 0.676 | 163.5±8.1 | 164.4±6.1 | 163.1±7.0 | 0.006 |
| BMI (kg/m^2^) | 25.1±4.0 | 24.7±4.1 | 24.1±4.0 | 0.376 | 25.0±4.2 | 24.6±3.7 | 24.2±4.2 | 0.042 |
| PA |  |  |  |  |  |  |  |  |
| Low (%) | 90 (48.4) | 83 (44.1) | 88 (47.3) | 0.237 | 99 (53.2) | 91 (48.4) | 71 (38.2) | < 0.0001 |
| Medium (%) | 57 (30.6) | 67 (35.6) | 48 (25.8) |  | 61 (32.8) | 42 (22.3) | 69 (37.1) |  |
| High (%) | 39 (21.0) | 38 (20.2) | 50 (26.9) |  | 26 (14.0) | 55 (29.3) | 46 (24.7) |  |
| SES |  |  |  |  |  |  |  |  |
| Low (%) | 71 (38.2) | 89 (47.3) | 72 (38.7) | 0.032 | 87 (46.8) | 54 (28.7) | 91 (48.9) | < 0.0001 |
| Medium (%) | 62 (33.3) | 39 (20.7) | 64 (34.4) |  | 48 (25.8) | 62 (33.0) | 55 (29.6) |  |
| High (%) | 53 (28.5) | 60 (31.9) | 50 (26.9) |  | 51 (27.4) | 72 (38.3) | 40 (21.5) |  |

GI= glycemic index, SFA= Saturated fatty acid, PUFA= Polyunsaturated fatty acid, BMI = body mass index, SES= Socio-economic status

*Data were presented as mean± SD for continues variables or number (percent) for categorical variables.

**P-values are from one-way analysis of variance (ANOVA).

**Supplementary Table 2.** Tehranian women's dietary intake based on the quality of macronutrients.*

| variables | GI tertiles | | | P value** | SFA/ PUFA tertiles | | | P value |
| --- | --- | --- | --- | --- | --- | --- | --- | --- |
|  | T1 | T2 | T3 |  | T1 | T2 | T3 |  |
|  | ≤53.84 | 53.85 - 62.79 | ≥62.80 |  | ≤ 1.00 | 1.01- 1.37 | ≥1.38 |  |
|  | n=186 | n=188 | n=186 |  | n=186 | n=188 | n=187 |  |
| Energy | 1988.8±  709.9 | 2117.1±  853.7 | 2405.1±  864.3 | 0.003 | 1809.7±747.0 | 2284.4±816.8 | 2409.1±802.8 | 0.453 |
| Fiber | 5.2±3.1 | 5.7±  2.8 | 5.8±  2.6 | 0.787 | 4.5±2.8 | 6.0±2.8 | 6.0±2.7 | 0.165 |
| Iron | 17.2±  5.9 | 16.0±  7.0 | 18.2±  7.2 | 0.302 | 15.7±7.0 | 19.1±6.9 | 16.6±5.9 | 0.651 |
| Magnesium | 224.2±  108.2 | 246.1±  109.9 | 272.6±  95.3 | 0.002 | 207.7±92.8 | 262.6±108.4 | 272.1±105.9 | 0.060 |
| Zinc | 7.4±4.1 | 8.5±  4.0 | 9.2±  3.8 | 0.004 | 6.3±3.1 | 9.3±4.3 | 9.5±3.9 | 0.020 |
| Potassium | 2760.1±  1375.5 | 3037.9±  1424.7 | 3137.7±  1202 | 0.126 | 2448.3±1208.5 | 3211.6±1355.8 | 3268.9±1310.2 | 0.012 |
| Phosphorus | 990.9±  493.2 | 1153.7±  567.1 | 1281.9±  542.0 | <0.0001 | 931.9±525.4 | 1224.6±523.6 | 1267.5±532.1 | 0.096 |
| Calcium | 863.5±  362.0 | 904.8±  449.7 | 979.4±  439.7 | 0.032 | 738.1±325.4 | 951.0±387.0 | 1058.3±473.2 | 0.001 |
| Vitamin A | 929.5±  774.6 | 1252.0±  920.2 | 1236.2±  795.1 | 0.090 | 750.5±620.7 | 1300.7±899.8 | 1364.4±847.1 | 0.006 |
| Vitamin D | 1.0±1.3 | 1.3±  1.4 | 1.5±  1.3 | 0.001 | 0.8±1.2 | 1.3±1.2 | 1.7±1.4 | <0.0001 |
| Vitamin E | 12.8±  7.5 | 11.7±  6.3 | 13.2±  6.2 | 0.203 | 14.5±6.4 | 14.1±7.1 | 9.2±5.1 | <0.0001 |
| Vitamin K | 124.9±  81.8 | 136.9±  88.0 | 127.0±  76.3 | 0.129 | 100.9±73.5 | 144.6±83.2 | 142.9±82.2 | 0.100 |
| Vitamin C | 108.2±  67.8 | 132.4±  83.5 | 132.1±  91.3 | 0.456 | 105.1±87.0 | 132.2±84.1 | 135.0±71.4 | 0.145 |
| Vitamin B1 | 1.7±0.7 | 1.8±  0.9 | 2.2±  0.9 | 0.041 | 1.6±0.8 | 2.1±0.9 | 1.9±0.8 | 0.894 |
| Vitamin B3 | 20.3±  9.4 | 21.7±  10.2 | 24.5±  10.4 | 0.210 | 18.3±9.5 | 25.5±11.0 | 22.7±8.5 | 0.658 |
| Vitamin B5 | 4.5±2.4 | 5.2±  2.4 | 5.6±  2.3 | 0.005 | 4.2±2.4 | 5.4±2.4 | 5.6±2.2 | 0.106 |
| Vitamin B6 | 1.3±0.8 | 1.6±0.8 | 1.8±  0.8 | 0.009 | 1.3±0.9 | 1.8±0.9 | 1.7±0.7 | 0.541 |
| Vitamin B9 | 272.4±  166.2 | 294.2±  167.2 | 313.8±  138.2 | 0.647 | 258.5±174.3 | 314.2±162.1 | 306.8±130.5 | 0.680 |
| Vitamin B2 | 1.6±0.8 | 1.8±  0.9 | 2±0.8 | 0.021 | 1.4±0.8 | 1.9±0.8 | 2.0±0.8 | 0.002 |
| Vitamin B12 | 2.9±2.1 | 3.3±  1.8 | 3.7±  2.1 | 0.001 | 2.1±1.6 | 3.7±2.2 | 4.0±1.9 | <0.0001 |
| biotin | 12.0±  8.6 | 15.3±  9.5 | 16.9±  8.3 | 0.024 | 10.9±7.6 | 16.7±9.9 | 16.6±8.3 | 0.016 |
| Fruits | 273.2±  211.3 | 273.6±  200.6 | 261.5±  212.8 | 0.245 | 243.1±181.5 | 272.7±199.1 | 291.4±237.4 | 0.430 |
| Vegetables | 313.9±  238.9 | 321.3±  211.4 | 324.4±  196.1 | 0.154 | 273.3±231.4 | 342.1±212.7 | 343.3±195.1 | 0.375 |
| Grains | 491.4 ±156.0 | 519.0±  214.5 | 643.9±  262.3 | 0.002 | 577.8±250.5 | 598.8±229.0 | 523.2±224.7 | 0.007 |
| Rice, pasta,  legumes, potatoes | 230.9±  140.8 | 273.6±  165.1 | 351.5±  197.6 | <0.0001 | 265.0±184.4 | 295.9±164.2 | 294.3±178.8 | 0.470 |
| Meat,  poultry, eggs | 55.6±  56.8 | 76.6±  61.5 | 80.4±  70.4 | <0.0001 | 40.7±38.4 | 84.0±67.2 | 87.5±70.1 | 0.021 |
| Dairy | 327.2±  173.0 | 311.3±  234.6 | 366.8±  266.6 | <0.0001 | 277.9±185.9 | 320.2±188.0 | 408.9±281.9 | <0.0001 |
| Oils and fats | 29.3±  20.7 | 26.7±  18.5 | 28.1±  17.3 | <0.0001 | 24.1±14.2 | 30.9±19.7 | 29.0±21.4 | 0.273 |

GI= glycemic index, SFA= Saturated fatty acid, PUFA= Polyunsaturated fatty acid

*Data were presented as mean± SD.

**P-values are from one-way analysis of variance (ANOVA).

**Supplementary table 3.** The association between Tehranian women's anthropometric and lipid indices based on the amount of macronutrients.*

| variables | Carbohydrate tertiles (g) | | | P trend** | Protein tertiles (g) | | | P trend | Fat tertiles (g) | | | P trend |
| --- | --- | --- | --- | --- | --- | --- | --- | --- | --- | --- | --- | --- |
|  | T1 | T2 | T3 |  | T1 | T2 | T3 |  | T1 | T2 | T3 |  |
|  | ≤259.70 | 259.71 - 386.94 | 386.95≥ |  | ≤58.23 | 58.24 - 86.61 | ≥86.62 |  | ≤ 46.94 | 46.95 - 75.19 | ≥75.20 |  |
|  | n=186 | n=189 | n=185 |  | n=186 | n=187 | n=187 |  | n=186 | n=187 | n=187 |  |
| Obesity |  | | | | | | | | | | | |
| Model ^1^ | 1 | 0.97  (0.65-1.46) | 0.92  (0.61-1.39) | 0.707 | 1 | 0.41  (0.27-0.62) | 0.55  (0.37-0.83) | 0.008 | 1 | 0.37  (0.24-0.57) | 0.53  (0.35-0.80) | 0.027 |
| Model ^2^ | 1 | 1.79  (0.99-3.23) | 4.43  (1.63-12.06) | 0.004 | 1 | 0.52  (0.30-0.91) | 0.68  (0.30-1.52) | 0.519 | 1 | 0.48  (0.29-0.81) | 0.86  (0.40-1.85) | 0.554 |
| *New anthropometric indices* | | | | | | | | | | | | |
| BRI |  | | | | | | | | | | | |
| Model ^1^ | 1 | 0.50  (0.32-0.77) | 0.59  (0.38-0.92) | 0.026 | 1 | 0.39  (0.25-0.61) | 0.47  (0.30-0.74) | 0.003 | 1 | 0.43  (28-0.67) | 0.50  (0.32-0.78) | 0.019 |
| Model ^2^ | 1 | 0.73  (0.40-1.33) | 1.43  (0.52-3.97) | 0.539 | 1 | 0.65  (0.36-1.16) | 1.15  (0.50-2.65) | 0.444 | 1 | 0.75  (0.44-1.29) | 1.29  (0.58-2.87) | 0.198 |
| LAP |  | | | | | | | | | | | |
| Model ^1^ | 1 | 1.05  (0.67-1.65) | 1.97  (1.28-3.04) | 0.001 | 1 | 0.53  (0.34-0.80) | 0.45  (0.29-0.70) | < 0.0001 | 1 | 0.85  (0.56-1.30) | 0.60  (0.39-0.93) | 0.025 |
| Model ^2^ | 1 | 1.18  (0.62-2.22) | 1.96  (0.67-5.72) | 0.231 | 1 | 1.10  (0.61-2.02) | 0.83  (0.34-2.02) | 0.611 | 1 | 0.59  (0.34-1.03) | 0.71  (0.31-1.64) | 0.74 |
| *Blood lipid risk index* | | | | | | | | | | | | |
| CRI-1 |  | | | | | | | | | | | |
| Model ^1^ | 1 | 0.53  (0.35-0.81) | 0.55  (0.36-0.84) | 0.006 | 1 | 0.74  (0.49-1.12) | 0.51  (0.33-0.78) | 0.002 | 1 | 50  (0.33-0.75) | 0.45  (0.29-0.69) | 0.001 |
| Model ^2^ | 1 | 0.95  (0.51-1.77) | 2.44  (0.85-7.03) | 0.121 | 1 | 1.22  (0.68-2.19) | 1.39  (0.60-3.22) | 0.460 | 1 | 0.56  (0.33-0.97) | 0.37  (0.16-0.86) | 0.039 |
| AC |  | | | | | | | | | | | |
| Model ^1^ | 1 | 0.60  (0.37-0.96) | 1.03  (0.62-1.69) | 0.857 | 1 | 0.47  (0.29-0.77) | 0.80  (0.48-1.32) | 0.545 | 1 | 0.26  (0.16-0.43) | 0.86  (0.49-1.49) | 0.355 |
| Model ^2^ | 1 | 0.67  (0.34-1.32) | 1.64  (0.50-5.34) | 0.530 | 1 | 0.38  (0.20-0.74) | 0.56  (0.21-1.48) | 0.484 | 1 | 0.25  (0.13-0.48) | 0.56  (0.20-1.52) | 0.480 |
| AIP |  | | | | | | | | | | | |
| Model ^1^ | 1 | 0.20  (0.11-0.39) | 0.33  (0.17-0.66) | 0.006 | 1 | 0.27  (0.14-0.52) | 0.29  (0.15-0.55) | 0.001 | 1 | 0.15  (0.07-0.30) | 0.24  (0.11-0.50) | 0.015 |
| Model ^2^ | 1 | 0.19  (0.08-0.45) | 0.39  (0.10-1.46) | 0.191 | 1 | 0.35  (0.16-0.78) | 0.39  (0.13-1.13) | 0.250 | 1 | 0.20  (0.09-0.46) | 0.33  (0.10-1.01) | 0.962 |

*Data are presented as OR (95% CI)

**Obtained from Binary logistic regression

Model ^1^: crude.

Model ^2^: adjusted for age (year), marital status, physical activity (MET/h), Soci-economic status, total energy intake (kcal).
